# Influence of Soil Salinity on Selected Element Contents in Different *Brassica* Species

**DOI:** 10.3390/molecules27061878

**Published:** 2022-03-14

**Authors:** Michaela Zeiner, Iva Juranović Cindrić, Ivan Nemet, Karla Franjković, Branka Salopek Sondi

**Affiliations:** 1 Man-Technology-Environment Research Centre, School of Science and Technology, Örebro University, Fakultetsgatan 1, 70182 Örebro, Sweden; 2 Department of Chemistry, Faculty of Science, University of Zagreb, Horvatovac 102a, 10000 Zagreb, Croatia; ijuranovic@chem.pmf.hr (I.J.C.); inemet@chem.pmf.hr (I.N.); franjkovickarla6@gmail.com (K.F.); 3 Department of Molecular Biology, Ruđer Bošković Institute, Bijenička Cesta 54, 10000 Zagreb, Croatia; branka.salopek.sondi@irb.hr

**Keywords:** *Brassicaceae*, cabbage, kale, metal and metalloid uptake, salinity stress

## Abstract

Climate changes in coastal regions cause increased soil salinity, a well-known type of environmental stress for a high number of agricultural crop species, including Brassicaceae, whose growth and development, and consequently the crop quality and yield, are affected by salinity stress. The aim of the present study is to investigate the effect of salt stress on micro- and macro-element homeostasis in different *Brassica* crops. Kale (*Brassica oleracea* var. *acephala*), white cabbage (*B. oleracea* var. *capitata*) and Chinese cabbage (*B. rapa* ssp. *pekinensis*) were grown hydroponically and treated with 200 mmol/L sodium chloride for 24 h to mimic short-term salt stress. The contents of Al, Ca, K, Mg, Na, B, Ba, Cd, Co, Cr, Cu, Fe, Mn, Ni, Pb, Sr, V and Zn were determined in the roots and leaves of the salt-treated plants and corresponding controls by inductively coupled plasma atomic emission spectrometry and inductively coupled plasma mass spectrometry. While Al, Ca, K, Mg and Na were determined in the mg/g range, the contents of the other elements were found at the µg/g level. A statistical analysis of the obtained data showed that the applied salt treatment significantly influenced the single-element contents in different plant parts. The major elements Ca, K and Mg were mainly unaffected in the more-salt-tolerant kale and white cabbage under salinity stress, while K and Mg were significantly decreased in the more-sensitive Chinese cabbage. The levels of micro-elements were found to be species/variety specific. In general, potentially toxic elements were accumulated in the roots of salt-treated plants to a higher extent than in the corresponding controls.

## 1. Introduction

The growing population and trends towards replacing meat with vegetables in the daily diet result in the necessity to ensure there are sufficient plant-derived food resources for everyone. High harvest yield can be obtained when plants grow under optimal conditions. However, plants, as sessile organisms, are often exposed to unfavourable environmental conditions, such as high temperature, cold, drought and salinity (abiotic stress), and to the attack of micro-organisms, insects and herbivores, which cause biotic stress. Amongst the abiotic factors, salinity has been found to be the most significant stress limiting the productivity of agricultural crops [1]. Depending on the agricultural crop, the decrease in harvest yield has been reported to range from 50% to 80%, depending on crop species, and salinity levels up to 320 mmol/L have also been reported [2]. Already in the 1980s, 20% of total cultivated lands and 33% of irrigated agricultural lands have been estimated to be affected by high salinity. Considering an annual increasing rate of 10%, in 2050, half of the arable land could be salinised [3]. Especially in coastal Mediterranean regions with semi-arid and arid climates, the effect of rising soil salinity and the related consequences for agriculture are of importance [4]. Elevated salinity levels lead to changes in morphological, physiological and biochemical processes in plants. These effects, especially regarding reduced growth and lowered yield, have been investigated in recent years [5,6,7], alongside studies aiming to develop salt-tolerant species [1,4].

A large and diverse group of widely consumed vegetables is represented by the Brassicaceae family. The majority of the widely grown crops, such as cabbages, broccoli, cauliflower, kale etc., belong to the genus *Brassica*. These cruciferous vegetables are characterized by their content of glucosinolates, specialized metabolites responsible for the specific tastes and odours of this food. Furthermore, they are rich in dietary fibres, calcium, carotenoids (provitamin A), vitamin C and certain beneficial phytochemicals (polyphenolics) [8]. The ancient Romans and Greeks already consumed brassicas, which later, in the Middle Ages, were also used for medical purposes [9]. Due to their important role in the human diet and nutrition, these plants are investigated in order to obtain plants that are more suitable for saline and dry lands [1,4].

Since salinity also influences water and nutrient uptake via the hindrance of root growth through ionic toxicity and osmotic effects [10], the interaction of micro- and macro-elements, including metals and metalloids, with plants needs to be researched under this aspect. Essential elements, namely, carbon (C), nitrogen (N), potassium (K), sulphur (S), magnesium (Mg), chlorine (Cl), calcium (Ca), iron (Fe), zinc (Zn), manganese (Mn), sodium (Na), copper (Cu), boron (B) and molybdenum (Mo), play an important role in plant metabolism. Problems related to the deficiency of elements are thus common in agricultural crops [11,12]. Apart from these essential elements, other elements exhibit harmful effects even at very low concentrations, such as Cd, Pb and V. In the case of the uptake of essential and harmful elements by the same mechanisms, an elevated uptake of the latter lowers the uptake of the former. Metal uptake in plants depends on many factors. One important aspect is the metal load in the surroundings, i.e., the soil composition as well as the pollution of the respective site [13,14]. Each site is also characterized by its climatic conditions, whose changes can even influence the uptake in the same plants across different years [15]. Species-specific metal uptake and accumulation can be studied by analysing various plants grown in the same area in order to exclude other influencing parameters.

For agricultural crops, a sufficient supply of essential elements has to be ensured, whilst potentially toxic elements (PTEs) should be present only at very low levels. Climate changes and anthropogenic activities may change plant environment and soil composition, causing disturbances in the homeostasis of micro- and macro-elements in plants. The purpose of this research study is to investigate the influence of increased salinity on the uptake and accumulation of micro- and macro-elements in the roots and leaves of three Brassica crops (kale, white cabbage and Chinese cabbage). Elemental analyses were performed by inductively coupled plasma atomic emission spectrometry and inductively coupled plasma mass spectrometry. This information is not only relevant under a nutritional aspect but also for risk assessment studies and applicability to certain (agricultural) lands.

## 2. Results and Discussion

### 2.1. Plant Growth and Salinity Stress

The salinity level of 200 mmol/L applied to the nutritional solution was chosen because this concentration has been previously reported to cause severe effects in brassica crops depending on their natural salinity tolerance [16].

Regarding root growth and reduction in the seedling biomass, all investigated *Brassica* species have been found to be strongly affected by high salinity (200 mmol/L), but Chinese cabbage has been reported to appeare to be the most sensitive, followed by white cabbage, while kale has been stated to be the most tolerant. Root-growth inhibition equal to or even more than 90% has been described for all three plants, whilst the seedling biomass has been reported to have only been reduced by 40–55% [17]. Conversely, air anions [18] and electric stimulation have been found to promote the growth of leafy vegetables [19] in closed cultivation.

### 2.2. Figures of Merit of the Analytical Procedure

The analytical procedures for inductively coupled plasma atomic emission spectrometry (ICP-AES) as well as inductively coupled mass spectrometry (ICP-MS) were found to be applicable to the given analytical task and sample type. The spiking experiments led to recoveries ranging from 86% to 115% for the elements determined, proving the trueness of the methods. The precision, expressed as relative standard deviation (RSD), ranged from <1% up to 5.9%. Combining good trueness and precision showed acceptable accuracy of the applied procedures. All external calibration curves had R^2^ values beyond 0.997.

### 2.3. Elemental Contents in Salt-Treated Roots and Leaves Compared with the Corresponding Control

#### 2.3.1. General Findings

The contents of all the elements determined refer to the dry matter mass of the respective plant tissue (leaves or roots). The table below (Table 1) shows the results for kale leaves. For these samples, more elements (30 instead of 18) were determined than for all the other ones, due to the option to analyse selected samples using a second method, namely, ICP-MS. The effect of elevated salinity on plant growth is also indicated in the tables, with arrows marking statistically significant increases (↑) or decreases (↓), whilst no statistically significant differences are shown using a dash (−), based on paired *t*-tests using level of significance of 95% (see Section 3.3.).

The levels of the elements detected are in a range comparable to that given in the literature [20,21,22,23,24]. When using literature data, it is crucial to check how the data are presented, i.e., whether they refer to fresh mass or dried matter and to which mass (100 g or 1 kg) [22,24], or if they list the mass of a certain element considering the entire plant [19]; it is also necessary to check which growing conditions and climatic influences are considered [13,15]. Furthermore, the pollution background of the soil where the vegetables grow determines the elemental contents observed in the plants and needs to be considered when comparing data, such as in the Ethiopian study with sampling carried out in an industrial area [23] or in another study analysing an area with high impact of atmospheric deposition [25]. It has to be taken into account that all data from the present study are relative data (content, i.e., element mass per mass of dried plant material); thus, in order to see the total amount of metals and metalloids taken up by a plant, the entire harvested mass of the leaves needs to be considered. Furthermore, natural variations in mineral uptake and accumulation play a role and affect the obtained data [26]. For nutritional aspects, however, as well as for the current research question, the contents were sufficient for the evaluation of risk and/or benefits, as well as changes in the uptake behaviour.

In the following figures, the elemental patterns are shown for each investigated plant species and plant tissue (root, leaves); the results for the untreated (control) plants are displayed on the left-hand side, while the results for the treated plants grown under salinity stress are shown on the right-hand side. In order to allow a visual evaluation of the results to be conducted, sodium was excluded from all pie charts due to its high content in the treated plants, in which its content increased 10–50-fold.

**Figure 1:**
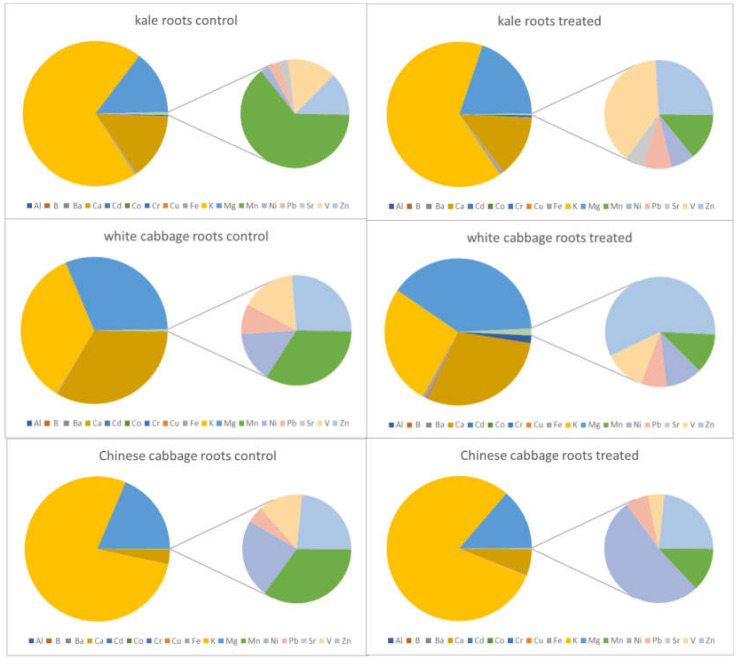
Elemental patterns of selected elements in the roots of treated and untreated plants without sodium.

**Figure 2:**
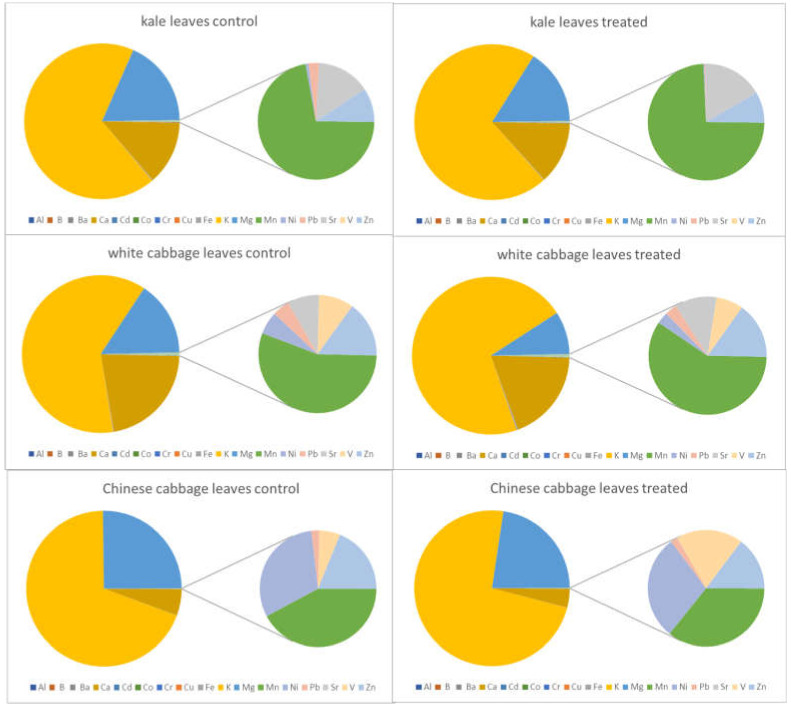
Elemental patterns of selected elements in the leaves of treated and untreated plants without sodium.

As it can be seen in Figure 1 and Figure 2, calcium, potassium and magnesium are the major elements found in the roots as well as in the leaves. All three *Brassica* species showed different ratios of these elements, and the differences were more evident in the roots than in the leaves.

The contents in the treated and untreated plant parts show no statistically significant differences for Ca, K, and Mg except for the roots of the Chinese cabbage, where a significant decrease was registered. Looking at the minor elements, especially the potentially toxic ones, are of higher interest for this study. Low bioconcentration factors have been reported for the species Chinese cabbage, namely less than 0.01 for As, Cr and Pb alongside 0.3 for Cd [27]. Cadmium, chromium, copper, nickel and vanadium were accumulated to a higher extent in the roots when the plants were exposed to elevated salt levels, regardless of the tested *Brassica* species. This tendency was particularly notable in white cabbage, but also in kale (compare Figure 1). Considering, however, that the root biomass was strongly reduced under the applied growing conditions, the total amount of the absorbed metals did not increase. Barium and cobalt showed the same behaviour as the above-listed elements, but only in kale, since their contents in the white cabbage and Chinese cabbage samples were below the respective limits of detection. For lead, another element that is harmful to the environment, wildlife and humans, similar trends were found in white cabbage and Chinese cabbage, i.e., an increase in the roots and no statistically significant differences in the leaves; on the other hand, in kale, no differences could be found in the roots, but there was a significant decrease in lead content in the edible parts. Aqueous extracts of Collard Green (*Brassica oleracea*) have been found to have a tendency for Pb uptake, removing more than 99% of Pb from contaminated water [28]. Aluminium, an element found in high abundance in the Earth’s crust, was found to behave differently in all three investigated plants. In white cabbage, Al showed the same pattern as other PTEs, namely, an increase in the roots and no changes in the leaves. However, in kale, Al was more highly accumulated in both roots and leaves. Chinese cabbage had even less Al in the roots when the plants were grown under salt stress, and no statistically significant changes were observed in the edible parts. Boron, an essential element for plants, but, nevertheless, not detected in the samples of Chinese cabbage, behaved similarly to Al, Cd, Cr, Cu, Ni, Pb and V in white cabbage; however, it was found to be present in kale leaves at lower levels, whilst its content in the roots was unaffected by higher salinity. Iron is another essential element for plants, and all three studied plants reacted differently to salt stress with respect to iron uptake and accumulation. Kale showed a response to Fe similar to that shown for B; in white cabbage, the content increased in the roots but significantly decreased in the leaves. In Chinese cabbage, Fe content was found to have changed similarly to the contents of the minor element Al and major elements K and Mg. Strontium content in roots and leaves was found not to be depending on salt stress; no statistically significant differences were registered in kale and white cabbage, whilst in Chinese cabbage, it was not present at levels above the LOD. Manganese, an essential element for plants as well as for humans, did not show significant changes due to elevated salinity in white cabbage but showed to have decreased in the roots and leaves of kale. In Chinese cabbage, only the edible parts were affected by salt stress, in such a way that less Mn was accumulated in the leaves, whilst untreated and treated roots had similar contents. Zinc contents were also found to have been affected by higher salinity in a species-dependent manner. Like for K, Mg and Sr, no statistically significant changes were observed for Zn in kale roots and leaves, as well aswhite cabbage leaves. Roots of white cabbage and Chinese cabbage, however. contained more Zn when treated with an elevated sodium chloride concentration. Conversely, Chinese cabbage leaves, showed a decrease in Zn content, whilst no differences were observed in white cabbage plants. The table below (Table 2) summarizes all these findings, which are based on paired t-tests and a decision level of *p* < 0.05 for the statistical significance of an observed change.

Even if studies of salt-stress effects on Brassicaceae species have been performed, they have mainly been focused on biomass and harvest yield but not on mineral contents, except for plant nutrients, e.g., sodium and potassium. A study of the levels of Ca, Cu, Fe, K, Mg, Mn, Na and Zn in three *Brassica* species treated with a NaCl solution at different concentrations ranging from 50 mmol/L up to 200 mmol/L showed similar values for Mg and Mn, but lower ones for the other elements [29]; this underlines the biological variation in the uptake behaviour of individual plants. Zhou and colleagues grew plants on soils enriched not only with sodium chloride but also with As, Cd, Pb and Zn, so that two parameters were changed making a comparison more complex [30]. A study of submersed plants showed that Pb content increased with salinity, whilst the contents of Cd, Cu and Zn decreased with the increase in the sodium chloride concentration [31]; however, the biological differences in aquatic plants and leafy vegetables adapted to other growing conditions have to be taken into account when comparing such studies. *Limonium brasiliense*, a coastal herb of the Plumbaginaceae family mainly found in Southern Brazil, was investigated for Pb uptake under salt stress. In this plant, the metal contents in the roots and aerial parts decreased with the elevation of the salinity level [32]. As the metal uptake can differ among various species of the same plant family, data for representatives of other families are only of limited use for comparison and data interpretation.

#### 2.3.2. Principal Component Analysis of Obtained Data

**Figure 3:**
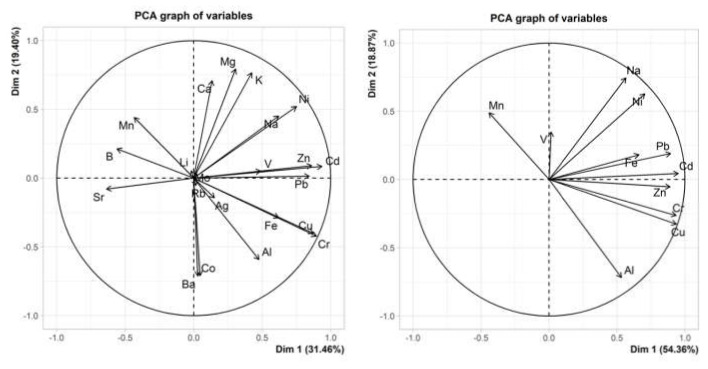
PCA graphs of variables for all elements (**left**) and only for minor elements (**right**) in the three *Brassica* species studied.

In addition to the paired *t*-tests performed individually for all elements and samples, the data were analysed by PCA. The graphs in Figure 3 show the contribution of each element, considering all analytes on the left-hand side and only the minor elements on the right-hand side. Whilst PC2 was more or less unchanged for both scenarios, the reduction in the number of analytes increased the percentage of PC1. Excluding the major elements, principal component 1 (PC1) and principal component 2 (PC2) carried 54% and 19% of the variance of the data, respectively.

**Figure 4:**
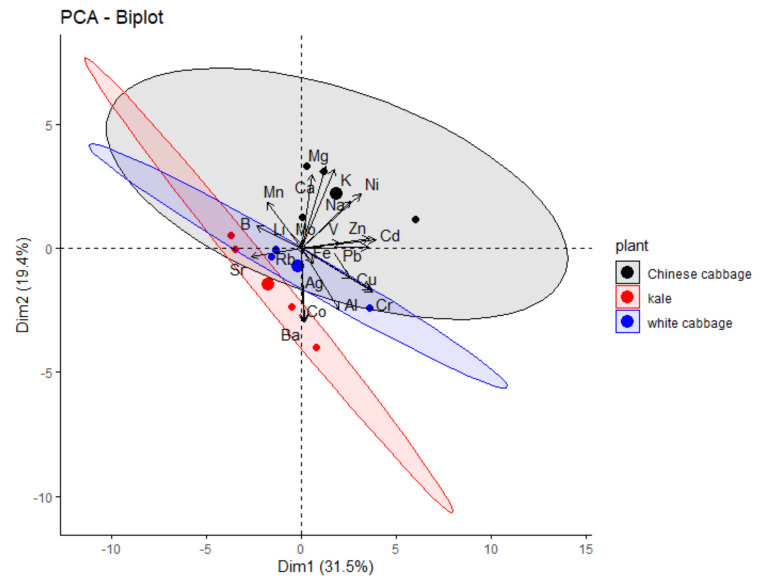
PCA biplot of elemental contents in the three Brassicaceae crops studied.

As already mentioned above, not only all three *Brassica* species showed distinct reactions to salinity but they also showed different uptake and accumulation patterns of the elements investigated, in agreement with their different salinity tolerances. This can be visualized in the PCA biplot below (Figure 4).

**Figure 5:**
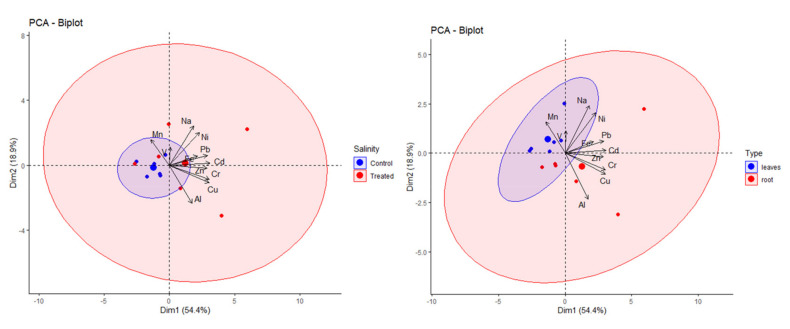
PCA biplots of elemental contents in the three Brassicaceae species studied with respect to salt treatment (**left**) and plant part (**right**).

Figure 5 shows the elemental contents in the three Brassica crops studied with respect to salt treatment (left) and plant organs (right). In particular, most treated samples appeared in the right part of the biplot, and their position relative to the loadings suggests that they had higher contents of most elements than the controls. Similarly, the contents found in the roots generally seemed to be higher than those found in leaves, since root samples generally appeared in the right part of the biplot.

## 3. Materials and Methods

### 3.1. Growing Plants and Sampling

Chinese cabbage (*B. rapa* L. ssp. *pekinensis* (Lour.) Hanelt cv. Cantonner Witkrop), white cabbage (*B. oleracea* var. *capitata* cv. Varaždinski) and kale (*B. oleracea* var. *acephala* cv. IJK9) were selected for the present investigation. Plants were grown hydroponically starting from seeds purchased from ISP International Seed Processing GmbH (Quedlinburg, Germany), Agricultural Advisory Service of Varaždin Region (Croatia) and Institute for Adriatic Crops and Karst Reclamation (Split, Croatia), respectively. After having been germinated on 1% agar plates, several-day-old seedlings were transferred into a hydroponic growth system with 5.5 L dark pots, as previously described [16]. Two nutrient solutions, namely, Flora Series and GHE Hydroponics, were purchased and used according to the manufacturers’ instructions. The conditions applied to the growing chamber were a temperature of 21 °C and a 16/8 h light (photons: 115 μmol/m^2^s)/dark cycle. From three to four weeks later (depending on the species), when the four fully developed leaves stage was reached, the plants were exposed to salinity stress by stepwise increasing the sodium chloride concentration in the nutrient solution until it finally reached 200 mmol/L (4–25 mmol/L, 2–50 mmol/L at 2 h intervals), whilst the nutrient solution of the control plants remained unchanged. Twenty-four hours after starting salinization, treated and untreated plants (controls) were harvested, and their leaves and roots were separated and quickly frozen using liquid nitrogen. All plant samples were then freeze-dried and stored before further analyses. The plant growing was carried out in four replicates (four separate pots), with each replicate consisting of eight plants per pot.

### 3.2. Sample Preparation and Elemental Analysis

The freeze-dried plant material was homogenized with a metal-free mortar prior to acidic microwave-assisted digestion. This sample preparation procedure was performed using an MWS-2 Microwave System Speedwave Berghof device applying the following temperature program: step 1—20 min, 500 W, 120 °C; step 2—30 min, 600 W, 170 °C; step 3—30 min, 400 W, 110 °C. Aliquots of 100–200 mg of dried matter were mixed with 5.0 mL of nitric acid (7 mol/L; diluted from 69% *w*/*w* HNO_3_ purchased from Kemika, Zagreb, Croatia) and 3.0 mL of hydrogen peroxide (1 mol/L; diluted from 30% *w*/*w* H_2_O_2_ purchased from Kemika, Croatia). The clear digests were filled to a final volume of 25 mL prior to analysis using inductively coupled plasma optical emission spectrometry (ICP-OES; Prodigy High Dispersive ICP). The digests of kale leaves were also analysed by inductively coupled plasma mass spectrometry (ICP-MS; Agilent 7500cx ICP-MS) after further dilution at 1:10 using 1% nitric acid (obtained from 65% *w*/*w;* Merck, Darmstadt, Germany) to increase the number of analytes. Digestion blanks were prepared in the same way and measured on the same day alongside the sample digests. The instrumental conditions for ICP-OES as well as for ICP-MS are listed in Table 3. For both methods, external calibration based on multi-elemental standard solutions prepared from ICP multi-element standard solution VI (Merck, Germany) was used for quantification. This mixed standard solution was also used to prepare spiked digest solutions (at two different concentrations) to check the precision and the trueness of the measurements conducted according to both methods.

### 3.3. Data Evaluation and Statistical Tests

All raw data obtained with the respective instrument (mass concentrations of the elements) were converted, considering digestion blank, final volume, dilution step and mass of sample digested, into contents in mg/kg of all analytes. Mean and standard deviation were calculated for each sample. The statistical significance of the differences found in elemental contents in the treated and control samples was individually verified for all elements and samples using paired t-tests. Additionally, a PCA (Principal Component Analysis) was carried out to ascertain the influence of salt treatment on the plants, alongside the contribution of each element analysed. For all tests, a level of significance of 95% was used for decision making. Microsoft Office Excel, v2016, and R 4.03 were used to perform the above-described calculations.

## 4. Conclusions

The investigation of the effects of salinity stress on important vegetables consumed by humans is of great and rising importance due to the ongoing salinization of arable land, especially in Mediterranean areas. Crop yield and quality can be significantly affected by increased soil salinity. This also includes the changes in the contents of essential as well as potentially toxic elements. Thus, three common *Brassica* crops (kale, white cabbage and Chinese cabbage) were grown under elevated salinity and analysed for their elemental contents. Regarding the major elements Ca, K and Mg, the contents remained more or less unchanged in kale and white cabbage, while a statistically significant decrease in K and Mg was found in the roots of Chinese cabbage. In general, harmful elements were accumulated at a higher level in the salt-treated plants than in the untreated controls and to a higher extent in the roots than in the edible parts (leaves). Essential minor elements, such as Fe and Zn, were not changed in the more-tolerant kale, while they were decreased in white cabbage and Chinese cabbage under salinity stress. Besides the drawback of reduced growth under high salt stress, the quality of the leafy parts did not change significantly when considering the mineral content, particularly in crops that were more tolerant to salinity stress.

## Figures and Tables

**Table 1:** Contents of selected elements in treated and untreated kale leaves, all values given in mg/kg.

Element	Control Leaves	Treated Leaves	Change *
Ag	<LOD	<LOD	
Al	20.8	52.8	↑
As	<LOD	<LOD	
B	79.3	52.8	↓
Ba	LOD < × < LOQ	LOD < × < LOQ	−
Be	<LOD	<LOD	
Bi	LOD < × < LOQ	LOD < × < LOQ	
Ca	8111	8144	−
Cd	0.090	0.044	↓
Co	0.091	0.108	−
Cr	2.09	1.03	↓
Cu	5.97	5.78	−
Fe	101	101	−
Ga	<LOD	<LOD	
K	41,572	44,850	−
Li	0.227	0.079	↓
Mg	11,124	10,057	−
Mn	205	185	↓
Mo	25.2	16.1	↓
Na	1665	65,752	↑
Ni	2.28	0.773	↓
Pb	8.34	0.672	↓
Rb	7.97	8.81	↑
Se	<LOD	<LOD	
Sr	42.5	41.9	−
Te	<LOD	<LOD	
Tl	<LOD	<LOD	
U	<LOD	<LOD	
V	0.043	0.038	−
Zn	27.2	21.5	−

* ↑ statistically significant increase; ↓ statistically significant decrease; – no statistically significant differences.

**Table 2:** Comparison table with changes in elemental contents caused by salinity.

Element	Kale		White Cabbage		Chinese Cabbage	
	Roots	Leaves	Roots	Leaves	Roots	Leaves
Al	increase	increase	increase	no significant changes	decrease	no significant changes
B	no significant changes	decrease	increase	no significant changes	<LOD	<LOD
Ba	increase	no significant changes	<LOD	<LOD	<LOD	<LOD
Ca	no significant changes	no significant changes	no significant changes	no significant changes	no significant changes	no significant changes
Cd	increase	decrease	increase	no significant changes	increase	no significant changes
Co	increase	no significant changes	<LOD	<LOD	<LOD	<LOD
Cr	increase	decrease	increase	no significant changes	increase	no significant changes
Cu	increase	no significant changes	increase	no significant changes	increase	no significant changes
Fe	increase	no significant changes	increase	decrease	decrease	no significant changes
K	no significant changes	no significant changes	no significant changes	no significant changes	decrease	no significant changes
Mg	no significant changes	no significant changes	no significant changes	no significant changes	decrease	no significant changes
Mn	decrease	decrease	no significant changes	no significant changes	no significant changes	decrease
Na	increase	increase	increase	increase	increase	increase
Ni	increase	decrease	increase	no significant changes	increase	no significant changes
Pb	no significant changes	decrease	increase	no significant changes	increase	no significant changes
Sr	no significant changes	no significant changes	no significant changes	no significant changes	<LOD	<LOD
V	increase	no significant changes	increase	no significant changes	increase	no significant changes
Zn	no significant changes	no significant changes	increase	no significant changes	increase	decrease

**Table 3:** Instrumental conditions for both analytical methods used.

Parameter	ICP-OES *	ICP-MS **
Instrument	Prodigy High Dispersive ICP-OES (Teledyne Leeman, Hudson, NH, USA)	Agilent 7500cx ICP-MS (Agilent, Tokyo, Japan)
Output power	1100 W	1500 W
Argon flows	Coolant: 18 L/min	Coolant: 15 L/min
	Auxiliary: 0.8 L/min	Auxiliary: 0.9 L/min
	Nebuliser: 1 L/min	Nebuliser: 0.2 L/min
Sample flow	1.0 mL/min	0.3 mL/min
Nebuliser	Pneumatic (glass concentric)	MicroMist
Spray chamber	Glass cyclonic	Scott double pass
Collison cell	-------	off

* at Department of Chemistry, Faculty of Science, University of Zagreb. ** at Man-Technology-Environment Research Centre, School of Science and Technology, Örebro University.

## Data Availability

Data, associated metadata and calculation tools are available from the corresponding author.
